# Staging LaParoscopy to Assess Lymph NOde InvoLvement in Advanced GAstric Cancer (POLA)—Study protocol for a single-arm prospective observational multicenter study

**DOI:** 10.1371/journal.pone.0285758

**Published:** 2023-05-19

**Authors:** Karol Rawicz-Pruszyński, Katarzyna Sędłak, Zuzanna Pelc, Radosław Mlak, Jakub Litwiński, Paweł Mańko, Krzysztof Zinkiewicz, Iwona Paśnik, Katarzyna Cięszczyk, Timothy Pawlik, Bruno Märkl, Maria Erodotou, Wojciech P. Polkowski

**Affiliations:** 1 Department of Surgical Oncology, Medical University of Lublin, Lublin, Poland; 2 Body Composition Research Laboratory, Department of Preclinical Sciences, Medical University of Lublin, Lublin, Poland; 3 Department of Clinical Pathomorphology, Medical University of Lublin, Lublin, Poland; 4 Department of Surgery, The Ohio State University Wexner Medical Center and James Cancer Center, Columbus, Ohio, United States of America; 5 Institute of Pathology, Klinikum Augsburg, Augsburg, Germany; 6 Department of Surgery, Erasmus MC, Rotterdam, The Netherlands; University of L’Aquila, ITALY

## Abstract

**Introduction:**

In the era of neoadjuvant chemotherapy in advanced gastric cancer (GC), the role of staging laparoscopy (SL) will become more established. However, despite guidelines recommendations, SL for optimal preoperative staging remains underutilized. Diagnostic value of near-infrared (NIR) / indocyanine green (ICG) guided sentinel node (SN) mapping in GC confirmed its technical feasibility, however no data exist regarding its potential role in pathological nodal staging. To the best of our knowledge, current study is the first to evaluate the role of ICG in nodal staging of advanced GC patients undergoing SL.

**Materials and methods:**

This single-arm prospective observational multicenter study was approved by the Bioethical Committee of Medical University of Lublin (Ethic Code: KE-0254/331/2018). The protocol is registered at clinicaltrial.gov (NCT05720598), and the study results will be reported according to the Strengthening of Reporting of Observational Studies in Epidemiology (STROBE) statement. The primary endpoint of this study is the identification rate of ICG-guided SN in advanced GC patients. The secondary endpoints include pathological and molecular assessment of retrieved SNs and other pretreatment clinical variables potentially associated with SL: pattern of perigastric ICG distribution according to patients’ pathological and clinical characteristics, neoadjuvant chemotherapy compliance, 30-day morbidity, and mortality.

**Conclusion:**

POLA study is the first to investigate the clinical value of ICG-enhanced sentinel node biopsy during staging laparoscopy in advanced GC patients in a Western cohort. Identifying pN status before multimodal treatment will improve GC staging process.

## Introduction

Recent discoveries in genetics, surgery and targeted therapies have continuously modified the well-established treatment protocol for gastric cancer (GC) patients [1]. In the locally advanced setting, multimodal therapy based on perioperative systemic treatment and surgery has been advocated for over a decade [2]. A patient-tailored treatment should rely on an effective staging process [3]. However, conventional diagnostic imaging modalities (CT, EUS, MRI) present limited accuracy [4], particularly in nodal staging (cN) [5]. At the same time, lymph node (LN) involvement is the only surgeon-dependent predictive factor in multimodal setting [6], which reflects the clinical significance of nodal staging.

Patients with pretreatment suspicion of LN metastases and good pathological response to multimodal treatment (ypN0) presents a comparable prognosis to clinically node-negative patients (cN0) [7]. Thus, the clinical and pathological nodal response should be the principal goal of preoperative therapy in cN+ patients [8]. Comprehensive lymph node assessment is critical for proper treatment strategy and survival prediction. Recent data on sentinel node (SN) concept [9] in GC has shown favorable results regarding LN detection rate and clinical status determination [10].

Hematoxylin and eosin (H&E) staining has been the gold standard for LN examination. However, these conventional pathological techniques are time-consuming, with final results available only several days after surgery. Therefore, a rapid, objective, and quantitative LN metastases assessment method is warranted [11]. One-step nucleic acid amplification assay (OSNA) is an in vitro diagnostic molecular assay system designed to assess the amount of cytokeratin 19 (CK19) messenger RNA (mRNA) within LNs [12]. The sum of all CK19 mRNA copies in analyzed LN is defined as total tumor load (TTL) [13]. Pooled data suggest that the OSNA assay has a high diagnostic accuracy for detecting LN metastases [12]. Recently, the effectiveness of ex vivo SN mapping with OSNA was compared with conventional histology, including immunohistochemistry in European GC patients [14]. Despite a relatively low detection rate (79%), OSNA SN evaluation showed high sensitivity, specificity, and accuracy rates of 85.4%, 93.5%, and 92.4%, respectively.

Staging laparoscopy (SL) with lavage cytology provides an additional value to clinical staging of GC [15–18], particularly in detecting occult peritoneal disease [19]. The yield of SL ranges between 7.8 and 53.4% [20], which reflects a lack of standardized and objective indications for the procedure. In the era of neoadjuvant chemotherapy (NAC), the role of SL will become more established. However, despite guidelines recommendations, SL for optimal preoperative staging remains underutilized [21].

A meta-analysis evaluating the diagnostic value of near-infrared (NIR) / indocyanine green (ICG) guided SN mapping in GC [22] confirmed its technical feasibility. ICG can be safely used for the identification of SN [23], determining the surgical resection line [24], improving the LN harvest, and reducing noncompliance in patients undergoing D2 lymphadenectomy [25].

The majority of the NIR/ICG studies in GC focused on the aspect of LN harvest optimization. At the same time, no data exist regarding its potential role in pathological nodal staging. To the best of our knowledge, current study is the first to evaluate the role of ICG in nodal staging of advanced GC patients undergoing SL.

## Materials and methods

### Objective

POLA study aims to investigate the safety and feasibility of ICG-guided SL with SN biopsy in advanced GC patients undergoing multimodal treatment. The pretreatment clinical variables potentially associated with the procedure will also be analyzed.

### Study design and setting

**Fig 1:**
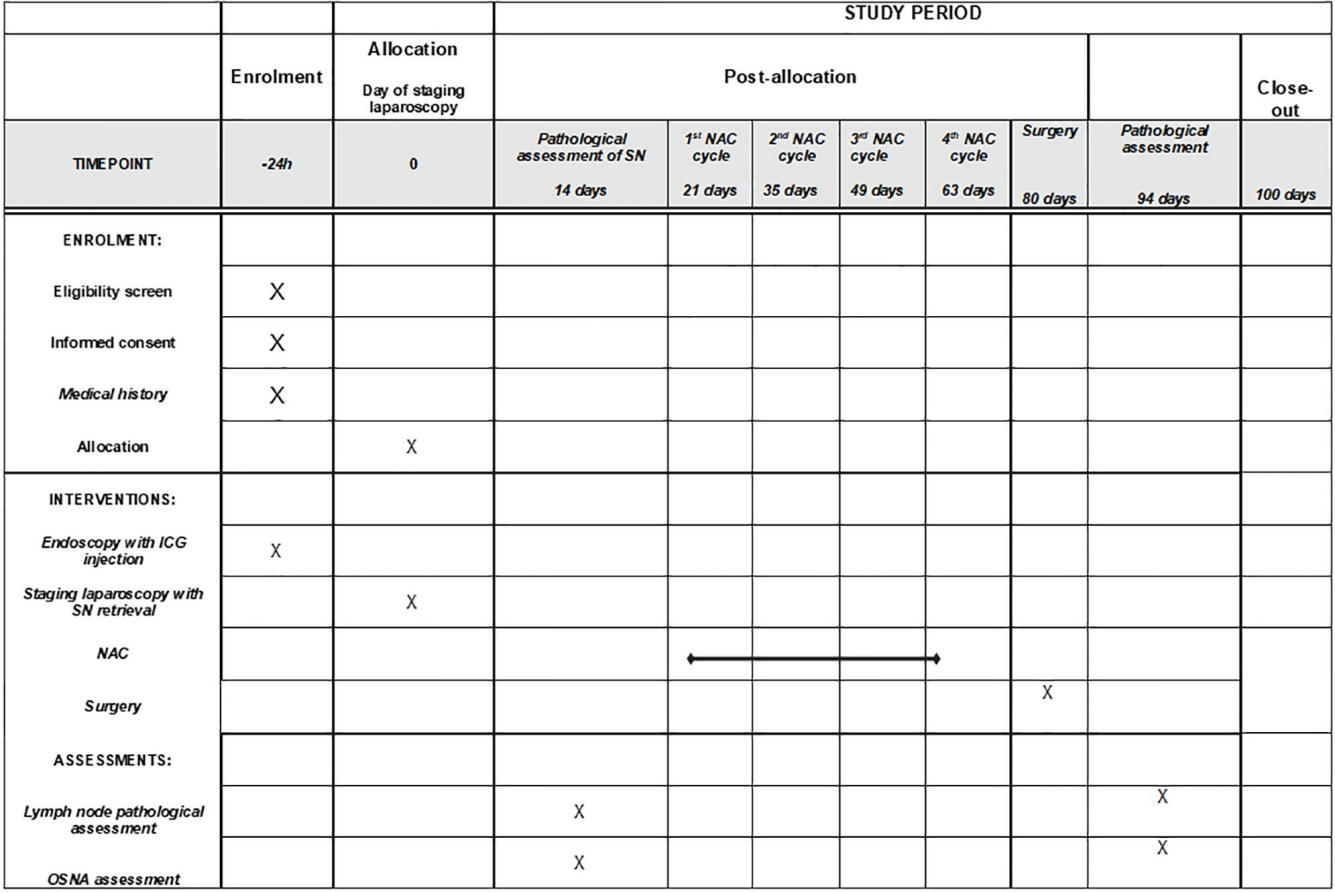
SPIRIT schedule for POLA study. ICG—Indocyanine green; SN—sentinel node; NAC—neoadjuvant chemotheraphy; OSNA—One-step Nucleid Acid Amplification.

This single-arm prospective observational multicenter study was approved by the Bioethical Committee of Medical University of Lublin (Ethic Code: KE-0254/331/2018) on the 20th December, 2018. The protocol is registered at clinicaltrial.gov (NCT05720598), and the study results will be reported according to the Strengthening of Reporting of Observational Studies in Epidemiology (STROBE) statement [26]. All procedures performed in study involving human participants are in accordance with the ethical standards of the institutional and national research committee and with the 1964 Helsinki Declaration and its later amendments or comparable ethical standards. SPIRIT (Standard Protocol Items: Recommendations for Interventional Trials) schedule of enrolment, interventions, and assessments is depicted on Fig 1.

### Inclusion criteria

Patients will be assessed for eligibility to participate in this study after verifying the following criteria:

1. Age ≥ 18 years
2. Histologically confirmed gastric adenocarcinoma (or undifferentiated carcinoma)
3. Stage II–III disease (cT2-4a, N0-3, M0) based on the pretreatment CT and 8th edition of TNM classification
4. Qualification for SL by the decision of the multidisciplinary tumor board
5. Written informed consent for endoscopy and SL

### Exclusion criteria

1. Early GC (cT1N0-3M0) scheduled for endoscopic treatment by the multidisciplinary tumor board
2. Previous abdominal surgery which could interfere lymphatic basin of the stomach, including previous gastrectomy, endoscopic (sub)mucosal dissection
3. Distant metastasis (cM1) clinically apparent in pretreatment abdominal/pelvic CT
4. Technical inability to perform endoscopic ICG injection or ICG injection beyond submucosa
5. Visual inability to identify the SN during SL or visualisation of SN beyond LN stations 1–12
6. Positive cytology (cyt+) after SL
7. Other malignancies
8. History of allergy to iodine agents

### Endpoints

The primary endpoint of this study is the identification rate of ICG-guided SN in advanced GC patients. The secondary endpoints include pathological and molecular assessment of retrieved SNs and other pretreatment clinical variables potentially associated with SL: pattern of perigastric ICG distribution according to patients’ pathological and clinical characteristics, neoadjuvant chemotherapy compliance, 30-day morbidity, and mortality.

### Study procedures

#### Endoscopic submucosal ICG injection

Patients will undergo upper GI endoscopy one day before SL. The ICG powder (Verdye^®^ 5mg/ml, 25mg powder for solution, Diagnostic Green, Ascheim-Dornach, Germany) will be dissolved in sterile water, resulting in a 0.125mg/ml concentration. 2 milliliters of the solution will be injected in the submucosa of 4 peritumoral sites– 0.5ml for each site.

#### Staging laparoscopy with sentinel node identification and retrieval

After abdominal cavity insufflation to 12mm Hg through the Veress needle or Hasson technique, an optical trocar will be installed below the umbilicus. An additional 5-mm and 10-mm trocars will be installed in the right and left upper quadrants, respectively. The parietal peritoneum of the diaphragm, abdominal and pelvic wall will be thoroughly observed for dissemination and presence of ascites. The peritoneal carcinomatosis index (PCI) will be determined after a meticulous inspection of 12 abdominal regions [27]. A surgical biopsy will be taken for pathological evaluation of macroscopic seeding, and ascites will be retrieved for cytological analysis. Otherwise, peritoneal lavage with an injection of 100ml saline around the tumor area will be performed, followed by retrieval of at least 50ml sample for cytological and molecular assessment. Intraoperative application of ICG-enhanced vision will be accomplished with dedicated optical devices. Alternate usage of white light and ICG fluorescence mode will allow precise location and cT stage determination of primary tumor, followed by identification of SN and its corresponding LN station, according to JGCA guidelines [28]. Identified SN will be retrieved with a high-energy device, and the LN basin will be labelled with a magnetic clip. Visualisation of the primary tumor and sentinel lymph node station / basin with ICG, followed by its sharp and blunt dissection with high-energy device during SL is showed in Vid. 1.

#### Sentinel node assessment

The SN assessment will be conducted similarly to the method proposed by Märkl et al. (14) All LNs will be stored in a −80°C freezer, immediately after retrieval. Within 1 to 3 days, each LN will be individually measured and weighed. Small LNs (<5 mm in short diameter) will be bisected, and half of the node will be processed for histological evaluation while the remaining half will be used for OSNA analysis. For intermediate-sized LNs (5–10 mm), a middle slice of about 2 mm thickness will be cut out for the histology, and the remaining parts of the node will be processed by OSNA. In large LNs (>10 mm), at least two slices will be cut out for histology, and the remaining parts of the node will be analyzed by OSNA.

#### OSNA assessment

The OSNA analysis will be performed using the Sysmex RD-100i system (Sysmex Europe, Norderstedt, Germany). Preparation will be done according to the manufacturer’s instructions. A cutoff of 250 CK19 copies/μL will be used for differentiating between negative and metastatic LNs(14). Samples in which no LN structure could be confirmed histologically will be excluded from the data analyses.

**Fig 2:**
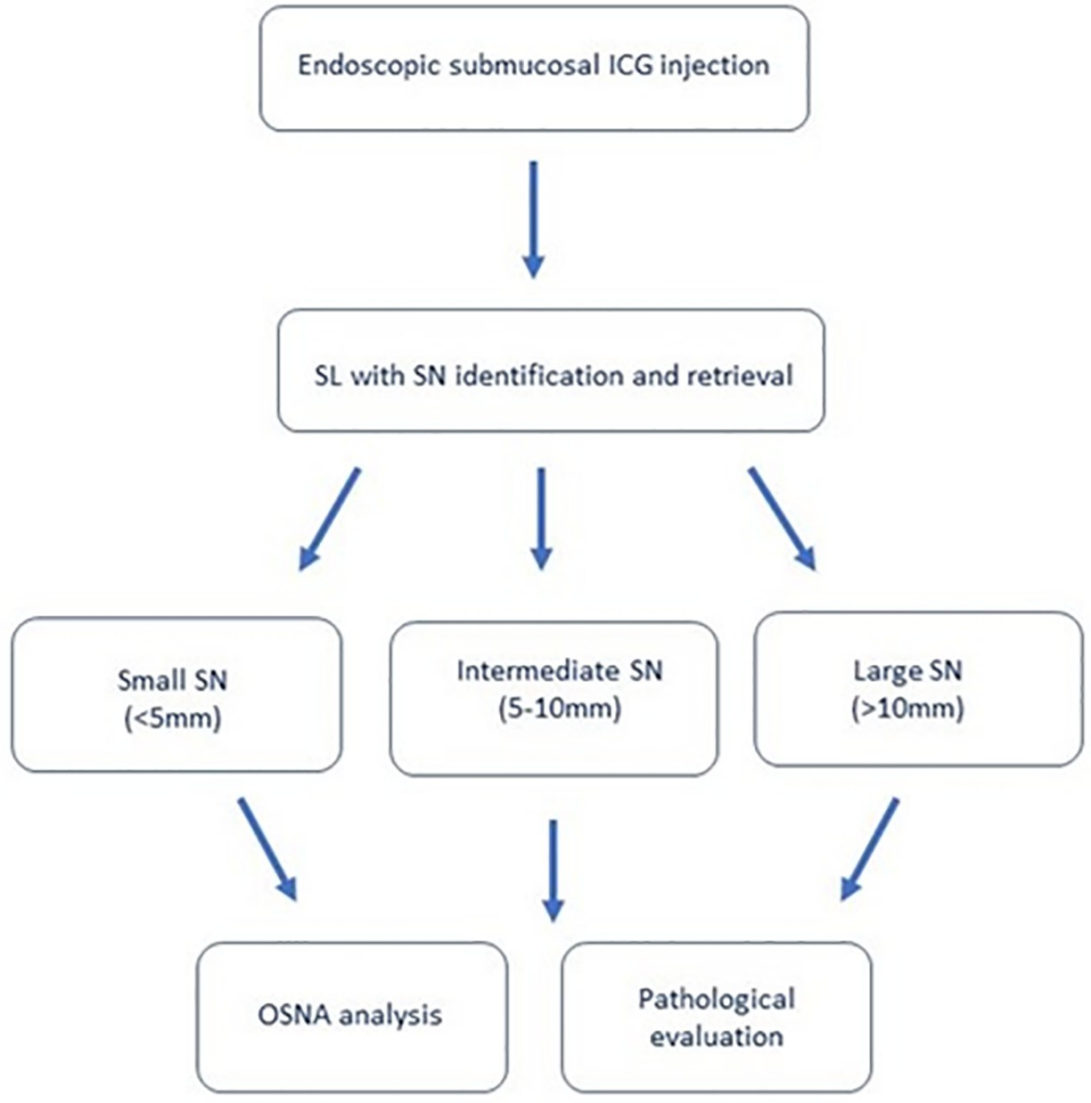
Flowchart of the study. Small SNs: bisection -> half of the SN processed for histological evaluation/remaining half of the nodule processed for OSNA analysis Intermediate SNs: middle slice (2 mm thickness) processed for histological evaluaton/remaining parts of the nodule processed for OSNA analysis Large SNs: ≥ 2 slices processed for histological evaluation/remaining parts of the nodule processed for OSNA analysis.

Flowchart of the study is depicted on Fig 2.

#### Neoadjuvant chemotherapy

NAC will be based on the Fluorouracil, Leucovorin, Oxaliplatin, and Docetaxel (FLOT) protocol, administered four cycles before and four cycles after the gastrectomy every two weeks. The regimen follows National Comprehensive Cancer Network (NCCN) guidelines [29]: docetaxel at 50 mg/ml, oxaliplatin at 85 mg/ml, leucovorin at 200 mg/ml and fluorouracil at 2600 mg/ml. Gastrectomy will be scheduled for at least four weeks after the last dose of NAC. In case of contraindication to docetaxel, the patients will be scheduled for FOLFOX (oxaliplatin 85 mg/m2, leucovorin 200 mg/m2, 5-FU bolus 400 mg/m2 and then 5-FU 2,400 mg/m2 as a continuous infusion over 46 h repeated every 2 weeks) or FLO (oxaliplatin at 85 mg/ml, leucovorin at 200 mg/ml and fluorouracil at 2600 mg/ml over 24 h each 2 weeks) regimen.

#### Gastrectomy

After obtaining written consent, patients will be scheduled for surgery performed by experienced surgeon with adequate LN dissection based on tumor pathology, size, and location. The "labelled" SN basin will be retrieved for pathological evaluation separately. The following perioperative surgical data will be registered:

- Type of surgery (open / laparoscopic / robotic)
- Extent of gastrectomy (total / proximal / distal)
- Method of reconstruction (Billroth I / Billroth II / Roux-en-Y / Double-Tract)
- Extent of lymphadenectomy (D1 / D1+ / D2 / D2+ / D3)
- Operative time
- Blood loss
- Comprehensive Complication Index
- Textbook Oncological Outcome (TOO)
- Additionally, a following histopathological data will be registered:
- Lauren histological type
- (y)pTNM
- Grading

### Statistical analysis

MedCalc v.15.8 (MedCalc Software, Belgium) will be used for statistical data analysis. D’Agostino-Pearson will be used to assess the normality of the data distribution. Depending on the continuous data distribution type, the mean and standard deviation or median and interquartile range / minimum-maximum range will be used as a measure of data concentration and spread (for normally and non-normally distributed data, respectively). Moreover, depending on the continuous data distribution type, parametric (t-test, Pearson’s correlation) or non-parametric tests (e.g., U-Mann-Whitney, Wilcoxon, Spearman’s correlation) will be used (for normally and non-normally distributed data, respectively). Categorized and dichotomized variables will be expressed as numbers and percentages. Chi-square or Fisher exact test will be used to assess the statistical difference in data distribution according to the studied groups. The test odds ratio (OR) and corresponding 95% confidence intervals will be used to assess the chance/risk of an occurrence of a particular phenomenon. Logistic regression models will be used in the multivariable analysis to assess chance/risk of an event of a specific phenomenon. Goodness-of-fit of obtained regression models will be validated via Hosmer-Lemeshow statistics. Overall survival (OS) will be defined as the time from the date of surgery to the date of patient death or the date of the last follow-up. The log-rank test will be used to calculate the proportional hazard ratio and the corresponding 95% CI in univariable OS analysis (the Kaplan-Meier estimation method will be used to generate survival curves), whereas Cox proportional hazard regression models will be used in multivariable OS analysis. In case of assessment of alternative methods of survival probability (e.g. due to problems with fulfilling Cox proportional hazard regression assumptions) accelerated failure time (AFT) models use will be considered. In all analyses, two-sided p-tests will be used, and results with a p-value below 0.05 will be considered statistically significant.

Since the character of the study is multicenter, participating departments will be compared with the use of the chi-squared test with Yates’ correction for continuity in terms of the primary endpoint before the main analysis. Assuming no statistically significant difference between centers, all will be included in the final analysis. Otherwise, for centers for which statistically significant differences will be noted, a separate analysis will be performed (after the prior enlargement of each study group to achieve the appropriate sample size).

### Sample size calculation

The calculation of the sample size was based on the primary endpoint of proposed study. Since there are no studies evaluating the usefulness of the ICG method in the identification of sentinel nodes (SN) in patients with advanced GC undergoing multimodal treatment, we calculated sample size based on 100% identification rate for this method in early GC [30, 31] and non-inferiority design. he margin of non-inferiority, defined as the largest difference that is clinically acceptable, was set to 5%. Most medical studies consider a p-value below 0.05 to reject the null hypothesis, thus type I error (alpha) of 0.05 value was used. In the case of type II error, we set a cut-off of beta on 0.2 to achieve 80% of statistical power. Accordingly, based on comparison of two independent proportions (100% and 95%, respectively) a sample size of 190 patients was considered appropriate for this single-arm study.

### Quality assurance

The quality assurance team associated with this study will include clinical oncology, oncological surgery, pathology, and radiology experts. Data censors will communicate with branch centers and randomly check the quality of data collection.

### Data collection and management

Each center will have at least two physicians to enrol patients in this study and arrange therapy during multidisciplinary team meetings. Two physicians will collect and secure data at their centers. All electronic documents will be confidential. The database will be under the project leader’s supervision, and no researcher will be allowed to use the data unless permitted.

## Discussion

Nodal involvement is one of the most critical, surgeon-dependent prognostic factors [32], while pathological response to NAC is an independent predictor of OS and 3-year disease-specific survival in GC patients [33, 34]. Despite multimodal treatment, the prognosis for ypN+ patients is poor, and recognising occult LN metastases in a preoperative setting remains challenging.

Machine learning (ML) algorithm is a newly emerged technique that aims to improve the effectiveness and applicability of pathological nodal staging in GC patients. A recent meta-analysis demonstrated that ML had an excellent diagnostic performance in predicting LN metastases [35]. Among 45 studies, a total of 56 182 patients have included, in which the number of patients with LN metastases was 12 031 (21.4%). However, only one study was performed in Europe, and none comprised patients after NAC.

In December 2022, 27 international experts agreed that fluorescence imaging with ICG is an acceptable single-agent modality for SN identification in GC, which has the potential to change GC surgery practice significantly [36].

Meta-analysis on the safety and efficacy of ICG in laparoscopic gastrectomy revealed that ICG tracer increases the LN harvest during radical D2 gastrectomy and databutes to the safety of conventional laparoscopic gastrectomy [37], including Western GC population [38]. However, it is undeniable that the role of ICG is limited to identifying LN, and pathological evaluation remains an essential component of nodal staging.

An ancillary study of the LOGICA trial aimed to investigate the pattern of metastases per LN station concerning tumor characteristics after D2 gastrectomy for GC patients undergoing multimodal treatment [39]. Although LN stations 3, 4, and 6 were involved most frequently (23%, 21%, and 22%, respectively), metastases were found in each LN station, regardless of tumor location, ycT-stage, Lauren histological subtype, and neoadjuvant chemotherapy. Authors indicated the need for further research to identify individual-tailored GC surgical treatment, particularly in the multimodal setting.

This study presents several potential limitations. Although SN identification will be based on the basin technique, as suggested by the expert above consensus [36], the possible presence of skip metastases may increase the false negative ratio of pN status. Secondly, implementation of ICG technology in GC surgery is steadily increasing, however its definite clinical value is yet to be established. Lastly, the technique of SL in GC patients requires standardization, and its role in nodal staging remains uncertain. Nonetheless, the latter issues are currently being addressed by authors of this study in an ongoing systematic review (CRD42022306746 in PROSPERO registry).

## Conclusion

POLA study is the first to investigate the clinical value of ICG-enhanced sentinel node biopsy during staging laparoscopy in advanced GC patients in a Western cohort. Identifying pN status before multimodal treatment will improve GC staging process.

## Supporting information

S1 Checklist SPIRIT 2013 checklist for POLA study. (DOCX)

S1 Dataset (XLSX)

S1 File (PDF)

S2 File (DOCX)
