# Supplementary material for: Staging LaParoscopy to Assess Lymph NOde InvoLvement in Advanced GAstric Cancer (POLA)—Study protocol for a single-arm prospective observational multicenter study
Source: PLoS One. 2023 May 19;18(5):e0285758. doi: 10.1371/journal.pone.0285758 (PMC10198545; doi:10.1371/journal.pone.0285758)
Supplement: S1 File — (PDF) [file pone.0285758.s003.pdf]

KOMISJA BIOETYCZNA [illegible]
[illegible] Al. Racławickie 1
20-059 Lublin, Al. Racławickie 1
tel. 81 448 52 13, fax 81 448 52 1[illegible]

UCHWAŁA KOMISJI BIOETYCZNEJ

numer KE-0254/331 /2018

W dniu **20 grudnia 2018 r.** Komisja Bioetyczna przy Uniwersytecie Medycznym w Lublinie, Al. Racławickie 1 zapoznała się z projektem badania:

„Badania nad poprawą efektywności procesu diagnostyczno – leczniczego u chorych na nowotwory układu pokarmowego oraz u chorych na raka piersi"

Projekt przedstawia:

prof. dr hab. Wojciech Polkowski
Klinika Chirurgii Onkologicznej
Uniwersytet Medyczny w Lublinie

Do Komisji wpłynęły następujące dokumenty:

Protokół badania
Informacja dla pacjenta
Formularz zgody pacjenta na udział w badaniu

Po zapoznaniu się z całością dokumentacji, zgodnie z zasadami GCP (Guidelines for Good Clinical Practice), Komisja Bioetyczna:

wyraziła **pozytywną** opinię o przedstawionym projekcie badania.

Niniejsza opinia traci moc z chwilą ukończenia badania.

KOMISJA BIOETYCZNA [illegible]

LISTA CZŁONKÓW KOMISJI BIOETYCZNEJ
opiniujących Uchwałę KE-0254/[illegible]/2018
z dnia 20 grudnia 2018 roku

Lp.	Imię i nazwisko	Zawód	Podpis
1.	dr hab. Marcin Olajossy	psychiatra	[redacted]
2	ks. prof. dr hab. Andrzej Szostek	duchowny	
3.	prof. dr hab. Jolanta Szymańska	stomatolog	
4	prof. dr hab. Paweł Krawczyk	internista	
5	prof. dr hab. Andrzej Dąbrowski	chirurg ogólny	
6	prof. dr hab. Wojciech Polkowski	chirurg onkologiczny	
7	dr hab. Elżbieta Czekajska – Chehab - prof. nadzw.	radiodiagnosta	
8	dr hab. Iwona Beń - Skowronek	pediatra	
9	dr hab. Janusz Kocki – prof. nadzw.	genetyk	
10	dr hab. Michał Skrzypek	internista, socjolog	
11	dr Hanna Czekajska - Łuckiewicz	farmaceuta	
12	dr Lidia Sierpińska	pielęgniarka	
13	mgr Stanisław Pęzioł	radca prawny	

PROTOKÓŁ BADANIA

TYTUŁ BADANIA:

Badania nad poprawą efektywności procesu diagnostyczno-leczniczego u chorych na nowotwory układu pokarmowego oraz u chorych na raka piersi

BADACZ:

Prof. dr hab. med. Wojciech P. Polkowski

OŚRODEK (OŚRODKI):

Uniwersytet Medyczny w Lublinie:

Klinika Chirurgii Onkologicznej UM w Lublinie

DOTYCZĄCE BADANIA NUMERY TELEFONÓW:

Klinika Chirurgii Onkologicznej UM w Lublinie:

81-534-43-13, 81-534-56-00

Wstęp

Rak piersi

U chorych na raka piersi, w trakcie zabiegu oszczędzającego gruczoł piersiowy najważniejszym celem chirurga jest wycięcie całego nowotworu. W marginesach cięcia nie powinna znaleźć się tkanka nowotworowa. Patolog ocenia marginesy cięcia oznaczone po zabiegu. W przypadku znalezienia komórek nowotworu w marginesach, chorej grozi nawrót miejscowy choroby i z tego powodu musi być ona poddana kolejnemu zabiegowi operacyjnemu. Niekiedy istnieje w takich przypadkach konieczność wykonania amputacji piersi. Nowotwór gruczołu piersiowego, szczególnie o niskim zaawansowaniu (obecnie najczęściej rozpoznawany w stadium T1c) rzadko jest dobrze odgraniczonym, jednorodnym guzem. Najczęściej ma nieostre kontury, promieniste wypustki i ogniska satelitarne, które nie są widoczne gołym okiem. Utrudnia to chirurgowi szybką ocenę doszczętności wykonanej

resekcji. Weryfikacja statusu regionalnych węzłów chłonnych pod kątem potencjalnych przerzutów pozostaje jednym z najistotniejszych czynników rokowniczych i predykcyjnych w raku piersi [1-3] Technologia OSNA znalazła swoje zastosowanie zarówno w śród- jak i pooperacyjnej analizie węzłów chłonnych wartowniczych (ang. Sentinel lymph node, SLN), których biopsja (ang. Sentinel node biopsy, SNB) jest złotym standardem u chorych, u których przedoperacyjnie nie stwierdza się klinicznie jawnych przerzutów w węzłach chłonnych pachowych. Jako wystandaryzowana metoda do precyzyjnej analizy węzłów wartowniczych, okazuje się być pomocnym narzędziem do podejmowania decyzji terapeutycznych, zarówno śródoperacyjnie (wskazania do limfadenektomii selektywnej), jak i podczas planowania terapii uzupełniającej [4-5]

1. Weaver DL i wsp. Effect of occult metastases on survival in node-negative breast cancer. N Engl J Med. 2011; 364(5):412–421

2. Fisher B i wsp. Relation of number of positive axillary nodes to the prognosis of patients with primary breast cancer. An NSABP update. Cancer. 1983; 52(9):1551–1557

3. Michaelson JS i wsp. The effect of tumor size and lymph node status on breast carcinoma lethality. Cancer. 2003; 98(10):2133–2143

4. Heilmann T i wsp. Intra-operative use of one-step nucleic acid amplification (OSNA) for detection of the tumour load of sentinel lymph nodes in breast cancer patients. J Cancer Res Clin Oncol. 2013; 139: 1649–55.

5. Deambrogio C i wsp. A new clinical cut-off of cytokeratin 19 mRNA copy number in sentinel lymph node better identifies patients eligible for axillary lymph node dissection in breast cancer. J Clin Pathol. 2014; 67(8): 702–6.

Rak żołądka

Jedyną skuteczną metodą leczenia raka żołądka pozostaje chirurgia, a jej skojarzenie z chemioterapią okołooperacyjną pozwala na wydłużenie czasu przeżycia chorych po operacji, jak również u chorych, u których stwierdzono obecność przerzutów odległych. Do przerzutów odległych zalicza się również przerzuty do poza-regionalnych węzłów chłonnych i otrzewnej. W leczeniu chirurgicznym, istotną rolę odgrywa zakres limfadenektomii. U chorych na zaawansowanego raka żołądka standardem jest wykonanie limfadenektomii D2, czyli usunięcie

węzłów chłonnych okołożołądkowych oraz węzłów wzdłuż tętnicy żołądkowej lewej, tętnicy wątrobowej wspólnej, pnia trzewnego i tętnicy śledzionowej aż do wnęki śledziony. Należy dążyć do tego, aby wynik badania histologicznego preparatu chirurgicznego po regionalnej limfadenektomii zawierał ocenę więcej niż 15 węzłów chłonnych [6].

Oprogramowanie WinEstimate służy do komputerowej oceny prawdopodobieństwa przerzutów do regionalnych węzłów chłonnych. Sumaryczne prawdopodobieństwo przerzutów w stacjach węzłów chłonnych, które nie zostały usunięte przez chirurga określa tzw. wskaźnik Maruyamy (ang. Maruyama index of unresected disease; MI) [7]. Śródoperacyjna ocena ryzyka za pomocą MI umożliwia indywidualne zaplanowanie zakresu limfadenektomii, jak również jest niezależnym czynnikiem rokowniczym u chorych na raka żołądka leczonych chirurgicznie oraz w skojarzeniu z radio-chemioterapią. Niska wartość MI (<5) jest związana z wydłużeniem przeżycia oraz mniejszym ryzykiem nawrotu choroby. Klinika Chirurgii Onkologicznej UM w Lublinie specjalizuje się w leczeniu skojarzonym chorych na raka żołądka, dlatego śródoperacyjna ocena obecności przerzutów do regionalnych i poza-regionalnych węzłów chłonnych za pomocą analizatora RD-100i jest uzasadniona naukowo i zwłaszcza w odniesieniu do praktyki klinicznej. Przerzuty raka żołądka do otrzewnej są najczęstszym powodem nawrotu choroby. Obecność wolnych komórek nowotworowych (ang. Free Cancer Cells- FCC) w płynie otrzewnowym jest wykorzystywana do przewidywania ryzyka nawrotu choroby i całkowitego przeżycia[8]. Rozsiew choroby do otrzewnej jest częstym powodem niepowodzenia po leczeniu chirurgicznym. Przerzuty do otrzewnej oraz obecność wolnego płynu w otrzewnej stwierdza się u 17% pacjentów poddanym resekcji z zamiarem leczniczym i wiążą się z pogorszeniem przeżycia [8] . Rozwój choroby w otrzewnej zależy nie tylko od stopnia jej zaawansowania, ale także od obecności FCC, które złuszczyły się z masy guza lub zajętych nowotworowo węzłów chłonnych. Komórki te mogą ulegać wszczepieniu do otrzewnej [8]. Zgodnie z ostatnią klasyfikacją TNM (8 edycja;2017) pacjenci, u których stwierdzono obecność FCC w płynie otrzewnowym kwalifikują się jako IV stadium choroby[9-10]. Ocena płynu otrzewnowego pod kątem FCC jest istotnym czynnikiem branym pod uwagę podczas podejmowania decyzji terapeutycznych [10]. Dokładniejsza analiza danych na tym etapie diagnostyki umożliwiałaby identyfikowanie chorych obarczonych wysokim ryzykiem wczesnego nawrotu choroby, co poza określeniem stopnia zaawansowania klinicznego choroby, umożliwiłoby szybsze zaplanowanie dalszych decyzji terapeutycznych.

marginesu resekcji. Badania w tym kierunku potencjalnie umożliwiłyby uzyskanie poprawy wyników odległych.

11 Borzomati, D i wsp. Microscopic residual tumor after pancreaticoduodenectomy: Is standardization of pathological examination worthwhile? Pancreas 2016; 45(5): 748–754.

12 Konstantinidis IT i wsp. Pancreatic ductal adenocarcinoma: is there a survival difference for R1 resections versus locally advanced unresectable tumors? What is a 'true' R0 resection? Annals of Surgery 2013; 257(4):731–736.

Etap kliniczny

Do badania zostaną zakwalifikowani pacjenci w wieku 18 – 85 roku życia z potwierdzonym histopatologicznie rakiem piersi lub nowotworem układu pokarmowego , którzy wyrazili i podpisali świadoma zgodę na udział w badaniu.

Opis pobranie płynu podczas laparoskopii lub operacji. Badaniu poddane będą krew (pobrana na surowicę, wyizolowana z próbek krwi rutynowo pobieranych do badań morfotycznych przed leczeniem operacyjnym u chorych leczonych w Klinice Chirurgii Onkologicznej) płyn otrzewnowy oraz materiał tkankowy z guza pobierany na wstępnym etapie leczenia operacyjnego. Pobrany płyn i/lub materiał tkankowy zostanie podzielony na dwie części i umieszczony w sterylnych opakowaniach. Jedna część płynu przeznaczona będzie do analizy cytologicznej w Zakładzie Patomorfologii SPSK1, druga część posłuży do oceny ekspresji CytK19 za pomocą systemu OSNA.

Etap Laboratoryjny

W Klinice Chirurgii Onkologicznej UM, analizie za pomocą systemu OSNA, poddana zostanie pozostała część płynu otrzewnowego i/lub materiału tkankowego. Płyn otrzewnowy / materiał tkankowy zostanie odwirowany w celu uzyskania osadu komórkowego. Otrzymane komórki będą przechowywane w temperaturze -80C do czasu wykonania analizy. Zastosowana technologia pozwoli na określenie liczby kopii genu CytK19, która koreluje z liczbą komórek

nowotworowych. Wyniki uzyskane za pomocą systemu OSNA, będą porównane do wyników uzyskanych za pomocą klasycznego badania cytologicznego / histopatologicznego

Wyniki

Prospektywne gromadzenie danych pozwoli na pro- i retrospektywne badania z zakresu nowotworów otrzewnej, w tym ocenę wczesnych i odległych wyników leczenia:

wyniki wczesne:

- powikłania (wg Dindo-Clavien oraz CCI)
- czas hospitalizacji pooperacyjnej (w tym na OIT)
- wskaźnik re-operacji
- śmiertelność pooperacyjna szpitalna
- śmiertelność 30-dniowa
- śmiertelność 90-dniowa

wyniki odległe:

- przeżycia całkowite (OS)
- przeżycia specyficzne dla choroby (DFS)

Wyniki badań zostaną opublikowane w indeksowanych czasopismach naukowych, oraz podczas krajowych i międzynarodowych zjazdów towarzystw chirurgicznych i onkologicznych

ZGŁOSZENIE EKSPERYMENTU MEDYCZNEGO
do Komisji Bioetycznej przy Uniwersytecie Medycznym
w Lublinie

1. Data złożenia zgłoszenia – 16.11.2018
2. Jednostka organizacyjna, w której będą wykonywane badania
   - Klinika Chirurgii Onkologicznej, Uniwersytet Medyczny w Lublinie

3. Kierownik samodzielnej jednostki organizacyjnej

   Prof. dr hab. Wojciech Polkowski, Kierownik Kliniki Chirurgii Onkologicznej, Uniwersytet Medyczny w Lublinie

3. Kierownik tematu badawczego (imię, nazwisko, stopień naukowy, specjalizacja)
   -Prof. dr hab. Wojciech Polkowski, Profesor Zwyczajny, Specjalista chirurgii ogólnej i onkologicznej
4. Członkowie zespołu badawczego (imiona, nazwiska, stopnie naukowe, specjalizacja, stanowisko służbowe)

   Uniwersytet Medyczny w Lublinie

   - Prof. dr hab. Wojciech Polkowski, Profesor Zwyczajny, Specjalista chirurgii ogólnej i onkologicznej, Kierownik Kliniki Chirurgii Onkologicznej, Uniwersytet Medyczny w Lublinie

   - Dr med. Jerzy Mielko, specjalista chirurgii ogólnej i onkologicznej, Adiunkt Kliniki Chirurgii Onkologicznej Uniwersytetu Medycznego w Lublinie,

   - Dr med. Andrzej Kurylcio, specjalista chirurgii ogólnej i onkologicznej, Adiunkt Kliniki Chirurgii Onkologicznej Uniwersytetu Medycznego w Lublinie

   - Dr med. Bogumiła Ciseł, specjalista onkologii klinicznej, Asystent w Klinice Chirurgii Onkologicznej Uniwersytetu Medycznego w Lublinie

   - Dr med. Magdalena Skórzewska, specjalista onkologii klinicznej, Asystent w Klinice Chirurgii Onkologicznej Uniwersytetu Medycznego w Lublinie

   - Dr med. Kinga Franciszkiewicz-Pietrzak, specjalista chirurgii ogólnej, Asystent w Klinice Chirurgii Onkologicznej Uniwersytetu Medycznego w Lublinie

   -Lek. Karol Rawicz-Pruszyński, Asystent w Klinice Chirurgii Onkologicznej Uniwersytetu Medycznego w Lublinie, w trakcie specjalizacji z chirurgii onkologicznej

- Lek. Magdalena Cisło, Rezydent w Klinice Chirurgii Onkologicznej Uniwersytetu Medycznego w Lublinie, w trakcie specjalizacji z onkologii klinicznej

-Mgr Katarzyna Gęca, Pracownik Naukowo-Techniczny w Klinice Chirurgii Onkologicznej Uniwersytetu Medycznego w Lublinie,

5. Temat badań (w języku polskim) – Badania nad poprawą efektywności procesu diagnostyczno-leczniczego u chorych na nowotwory układu pokarmowego oraz u chorych na raka piersi
6. Przewidywany termin zakończenia badań: bezterminowo
7. Informacja o charakterze prac, w ramach których prowadzone jest badanie (np.: licencjacka, magisterska, doktorska, inne) – realizacja zadań statutowych
8. Program badania, założenia, metody, opis grupy badawczej, liczebność, wiek, płeć, itp.)

Celem niniejszego badania jest poprawa efektywności procesu diagnostyczno-leczniczego u chorych na nowotwory układu pokarmowego oraz u chorych na raka piersi . Analiza ta umożliwi stworzenie kompleksowego algorytmu postępowania diagnostyczno-terapeutycznego w w.w nowotworach.

Do badania zostaną zakwalifikowani pacjenci w wieku 18 – 85 roku życia, u których rozpoznano pierwotny nowotwór złośliwy piersi lub nowotwór układu pokarmowego i którzy wyrazili zgodę na leczenie operacyjne w Klinice Chirurgii Onkologicznej Uniwersytetu Medycznego w Lublinie, oraz na udział w niniejszym badaniu. Prospektywne gromadzenie danych pozwoli na pro- i retrospektywne badania z zakresu nowotworów żołądka.

Wyniki badań zostaną opublikowane w indeksowanych czasopismach naukowych, oraz podczas krajowych i międzynarodowych zjazdów towarzystw chirurgicznych i onkologicznych
