# Supplementary material for: Staging LaParoscopy to Assess Lymph NOde InvoLvement in Advanced GAstric Cancer (POLA)—Study protocol for a single-arm prospective observational multicenter study
Source: PLoS One. 2023 May 19;18(5):e0285758. doi: 10.1371/journal.pone.0285758 (PMC10198545; doi:10.1371/journal.pone.0285758)
Supplement: S2 File — (DOCX) [file pone.0285758.s004.docx]

**Resolution of Bioethical Committee of Medical University of Lublin**

**No KE-0254/331/2018**

On 20th December 2018, the Bioethical Committee of the Medical University of Lublin became acquainted with the study protocol:

*“ Research on improving the effectiveness of staging and treatment of gastric cancer patients undergoing multimodal treatment”*

Presented by Prof. Wojciech Polkowski, Head of the Department of Surgical Oncology, Medical University of Lublin

After evaluating the study protocol under the Guidelines for Good Clinical Practice (GCP) regulations, the Bioethical Committee **approved** the study protocol.

The approval was signed by

**Head of the Committee – prof. Marcin Olajossy,**

and its members:

Prof Andrzej Szostek,

Prof. Jolanta Szymańska,

Prof Paweł Krawczyk,

Prof Beń-Skowronek,

Prof Kocki,

Prof. Skrzypek,

Prof. Sierpińska.

POLA study aims to investigate the safety and feasibility of indocyanine (ICG)-guided sstaging laparoscopy (SL) with sentinel node (SN) biopsy in advanced gastric cancer (GC) patients undergoing multimodal treatment. The pretreatment clinical variables potentially associated with the procedure will also be analyzed.

Patients will be assessed for eligibility to participate in this study after verifying the following criteria:

1. Age ≥ 18 years
2. Histologically confirmed gastric adenocarcinoma (or undifferentiated carcinoma)
3. Stage II – III disease (cT2-4a, N0-3, M0) based on the pretreatment CT and 8th edition of TNM classification
4. Qualification for SL by the decision of the multidisciplinary tumor board
5. Written informed consent for endoscopy and SL

And excluded from participation in the study when diagnosed with following criteria:

1. Early GC (cT1N0-3M0) scheduled for endoscopic treatment by the multidisciplinary tumor board
2. Previous abdominal surgery which could interfere lymphatic basin of the stomach, including previous gastrectomy, endoscopic (sub)mucosal dissection
3. Distant metastasis (cM1) clinically apparent in pretreatment abdominal/pelvic CT
4. Technical inability to perform endoscopic ICG injection or ICG injection beyond submucosa
5. Visual inability to identify the SN during SL
6. Positive cytology (cyt+) after SL
7. Other malignancies
8. History of allergy to iodine agents

The primary endpoint of this study is the identification rate of ICG-guided SN in advanced GC patients. The secondary endpoints include pathological and molecular assessment of retrieved SNs and other pretreatment clinical variables potentially associated with SL: pattern of perigastric ICG.

***Study Procedures***

*Endoscopic submucosal ICG injection*

Patients will undergo upper GI endoscopy one day before SL. The ICG powder (Verdye® 5mg/ml, 25mg powder for solution, Diagnostic Green, Ascheim-Dornach, Germany) will be dissolved in sterile water, resulting in a 0.125mg/ml concentration. 2 milliliters of the solution will be injected in the submucosa of 4 peritumoral sites – 0.5ml for each site.

*Staging Laparoscopy with Sentinel Node identification and retrieval*

After abdominal cavity insufflation to 12mm Hg through the Veress needle or Hasson technique, an optical trocar will be installed below the umbilicus. An additional 5-mm and 10-mm trocars will be installed in the right and left upper quadrants, respectively. The parietal peritoneum of the diaphragm, abdominal and pelvic wall will be thoroughly observed for dissemination and presence of ascites. The peritoneal carcinomatosis index (PCI) will be determined after a meticulous inspection of 12 abdominal regions (27). A surgical biopsy will be taken for pathological evaluation of macroscopic seeding, and ascites will be retrieved for cytological analysis. Otherwise, peritoneal lavage with an injection of 100ml saline around the tumor area will be performed, followed by retrieval of at least 50ml sample for cytological and molecular assessment. Intraoperative application of ICG-enhanced vision will be accomplished with dedicated optical devices. Alternate usage of white light and ICG fluorescence mode will allow precise location and cT stage determination of primary tumor, followed by identification of SN and its corresponding LN station, according to JGCA guidelines(28). Identified SN will be retrieved with a high-energy device, and the LN basin will be labelled with a magnetic clip.

*Sentinel Node Assessment*

The SN assessment will be conducted similarly to the method proposed by Märkl et al. All LNs will be stored in a −80 °C freezer, immediately after retrieval. Within 1 to 3 days, each LN will be individually measured and weighed. Small LNs (<5 mm in short diameter) will be bisected, and half of the node will be processed for histological evaluation while the remaining half will be used for OSNA analysis. For intermediate-sized LNs (5–10 mm), a middle slice of about 2 mm thickness will be cut out for the histology, and the remaining parts of the node will be processed by OSNA. In large LNs (>10 mm), at least two slices will be cut out for histology, and the remaining parts of the node will be analyzed by OSNA.

*OSNA assessment*

The OSNA analysis will be performed using the Sysmex RD-100i system (Sysmex Europe, Norderstedt, Germany). Preparation will be done according to the manufacturer's instructions. A cutoff of 250 CK19 copies/µL will be used for differentiating between negative and metastatic LNs. Samples in which no LN structure could be confirmed histologically will be excluded from the data analyses.

*Neoadjuvant chemotherapy*

NAC will be based on the Fluorouracil, Leucovorin, Oxaliplatin, and Docetaxel (FLOT) protocol, administered four cycles before and four cycles after the gastrectomy every two weeks. The regimen follows National Comprehensive Cancer Network (NCCN) guidelines(29): docetaxel at 50 mg/ml, oxaliplatin at 85 mg/ml, leucovorin at 200 mg/ml and fluorouracil at 2600 mg/ml. Gastrectomy will be scheduled for at least four weeks after the last dose of NAC. In case of contraindication to docetaxel, the patients will be scheduled for FOLFOX (oxaliplatin 85 mg/m2, leucovorin 200 mg/m2, 5-FU bolus 400 mg/m2 and then 5-FU 2,400 mg/m2 as a continuous infusion over 46 h repeated every 2 weeks) or FLO (oxaliplatin at 85 mg/ml, leucovorin at 200 mg/ml and fluorouracil at 2600 mg/ml over 24 h each 2 weeks) regimen.

*Gastrectomy*

After obtaining written consent, patients will be scheduled for surgery performed by experienced surgeon with adequate LN dissection based on tumor pathology, size, and location. The "labelled" SN basin will be retrieved for pathological evaluation separately.

*Statistical Analysis*

MedCalc v.15.8 (MedCalc Software, Belgium) will be used for statistical data analysis. D'Agostino-Pearson will be used to assess the normality of the data distribution. Depending on the continuous data distribution type, the mean and standard deviation or median and interquartile range / minimum-maximum range will be used as a measure of data concentration and spread (for normally and non-normally distributed data, respectively). Moreover, depending on the continuous data distribution type, parametric (t-test, Pearson's correlation) or non-parametric tests (e.g., U-Mann-Whitney, Wilcoxon, Spearman's correlation) will be used (for normally and non-normally distributed data, respectively). Categorized and dichotomized variables will be expressed as numbers and percentages. Chi-square or Fisher exact test will be used to assess the statistical difference in data distribution according to the studied groups. The test odds ratio (OR) and corresponding 95% confidence intervals will be used to assess the chance/risk of an occurrence of a particular phenomenon. Logistic regression models will be used in the multivariable analysis to assess chance/risk of an event of a specific phenomenon. Overall survival (OS) will be defined as the time from the date of surgery to the date of patient death or the date of the last follow-up. The log-rank test will be used to calculate the proportional hazard ratio and the corresponding 95% CI in univariable OS analysis (the Kaplan-Meier estimation method will be used to generate survival curves), whereas Cox logistic regression models will be used in multivariable OS analysis. In all analyses, two-sided p-tests will be used, and results with a p-value below 0.05 will be considered statistically significant.
